# Outcome of Continuous Positive Airway Pressure Adherence Based on Nasal Endoscopy and the Measurement of Nasal Patency—A Prospective Study

**DOI:** 10.3390/life13010219

**Published:** 2023-01-12

**Authors:** Zdeněk Knížek, Miloš Kotulek, Pavlína Brothánková, Eva Pecháčková, Pavel Klail, Tomáš Kostlivý, Jan Vodička

**Affiliations:** 1 Department of Otorhinolaryngology and Head and Neck Surgery, Hospitals of Pardubice Region—Hospital in Pardubice, 530 03 Pardubice, Czech Republic; 2 Faculty of Medicine in Hradec Králové, Charles University, 500 03 Hradec Králové, Czech Republic; 3 Faculty of Health Studies, University of Pardubice, 532 10 Pardubice, Czech Republic; 4 Department of Otorhinolaryngology, University Hospital in Pilsen, Faculty of Medicine, Charles University, 323 00 Pilsen, Czech Republic

**Keywords:** CPAP, flow measurement, nasal obstruction, OSA

## Abstract

The gold standard for treating obstructive sleep apnea in adults is continuous positive airway pressure (CPAP). However, it can be difficult to convince patients to adhere to this therapy. The aim of this study was to determine the relationship between nasal endoscopy findings/nose patency and CPAP adherence. **Material and methods:** A cohort of 450 consecutive patients suspected of having OSA were prospectively enrolled. For further analyses, 47 OSA patients undergoing CPAP treatment were selected (13 females and 34 males, average age, 65.3 years, BMI 34.1, apnea-hypopnea index. AHI 51.0). The patients were divided into two groups: patients with good CPAP adherence (*n* = 35) and patients who did not adhere to CPAP therapy (*n* = 12). The influence of nasal endoscopy and flow measurement on CPAP adherence was explored. **Results:** We found a statistical independence between adherence to CPAP and AHI (*p* = 0.124), T90 (*p* = 0.502), endoscopic findings (*p* = 0.588) and nasal patency measured by a flowmeter (*p* = 0.498). **Conclusions:** In our studied sample, endoscopic findings and nasal patency measured by a flowmeter were not predictors of CPAP non-adherence in the first year of the treatment. Our data show that while an endoscopic finding in the nasal cavity could indicate that a patient has a severe obstruction, compliance with CPAP therapy is not reduced in these patients and neither is it reduced with a decrease in nasal flow, according to our observation.

## 1. Introduction

Adult obstructive sleep apnea (OSA) is a sleep-related breathing disorder. Its diagnostic criteria according to the ICSD-3 are the presence of one or more of the following: the patient related daytime or nighttime symptoms (e.g., sleepiness, non-restorative sleep, waking with breath holding etc.) and/or bed-partner observations (snoring, breathing interruptions, etc.) and/or medical condition associated with OSA (hypertension, mood disorder, cognitive dysfunction, coronary artery disease, stroke, congestive heart failure, atrial fibrillation or type 2 diabetes mellitus) and at the same time a polysomnography (PSG) or an out-of-center sleep-testing (OCST) demonstrated five or more predominantly obstructive respiratory events per hour of sleep. Alternatively, the diagnosis is made when a PSG or an OCST demonstrates 15 or more predominantly obstructive respiratory events per hour of sleep [1]. 

Sleep-related disordered breathing is highly prevalent in our population. The HypnoLaus study which based its data on a cohort of 3043 patients, suggests a prevalence of moderate or severe OSA as high as 23.4% in women and of 49.7% in men [2]. The severity of OSA proportionately raises the risk for OSA-related comorbidities in multiple organ systems. OSA has been shown to contribute to cardiovascular, respiratory and neurologic impairments; notably, OSA and cardiovascular disease are strongly correlated [3,4]. 

The role nasal obstruction plays in the pathophysiology of OSA is yet to be fully elucidated. A number of pathophysiological mechanisms can potentially explain the role of nasal pathology in OSA. These include: the Starling resistor model, the unstable oral airway, the nasal ventilatory reflex and the role of nitric oxide [5]. Yet, we observe that the more severe the sleep-related breathing disorder, the less the patients tend to breathe through their mouth alone [6].

Continuous positive airway pressure (CPAP) remains the gold standard for the treatment of moderate or severe OSA in adults [7,8]; oral appliances and surgical treatment are preferred for milder forms of OSA [9,10]. Despite the efficacy of CPAP, many patients find it hard to adhere to this form of therapy [11,12]. Adherence to CPAP therapy is defined as using the therapy for at least four hours a day and for at least 70% of the nights in a year [13,14]. Among the most common reasons for CPAP failure are claustrophobia, mask discomfort, difficulties sleeping, an inability to keep the mask on, sensations of suffocating and nasal congestion. Nasal congestion has been reported as the cause of the failure to adhere to the therapy in multiple studies [15,16,17,18].

Despite the fact that nasal surgery itself does not reduce obstructive respiratory events, nasal surgery may reduce daytime and nighttime OSA-related symptoms (excessive daytime sleepiness, snoring) and may improve CPAP adherence [19,20,21,22,23,24]. 

According to several studies, the objective confirmation of the presence of a nasal obstruction can be used as a predictor of CPAP therapy non-adherence [25,26]. The aim of this study was to determine the impact of clinically significant nasal septal deformities and/or an inferior turbinates’ hypertrophy on CPAP therapy adherence. 

## 2. Material and Methods

### 2.1. Material

A total of 450 patients were enrolled in our prospective, monocentric, analytical study. The study ran from 6/2018 to 3/2021 in the tertiary referral hospital. Finally, a sample of 47 patients fulfilled the inclusion criteria and were included in the study. The study was approved by the Ethics Committee of the Hospitals of Pardubice region (reference number 6/2015). All the subjects signed a consent form before being enrolled in our study. 

**Figure 1:**
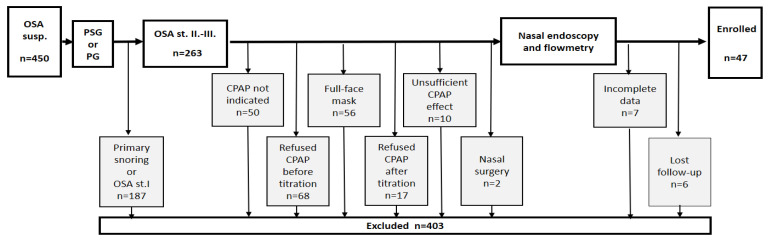
Flowchart of the inclusion/exclusion process.

The inclusion criteria were defined as: suspicion of OSA, sleep monitoring performed by PSG or limited polygraphy (PG), AHI ≥ 15, CPAP therapy with a nasal mask, being over 18 years of age. The exclusion criteria were: sleep monitoring performed by tools other than PSG or PG, nasal injury or prior surgery in the upper airways (patients who underwent adenectomy in childhood were not excluded), chronic disease of the paranasal sinuses, chronic pulmonary disease, CPAP therapy with a full-face mask, incomplete data and lack of cooperation. For details, see the flowchart in Figure 1.

**Figure 2:**
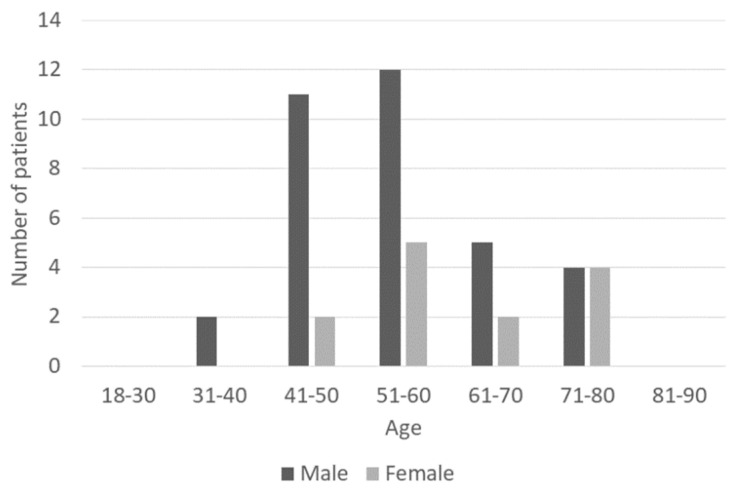
Age and gender distribution of the patients.

The selected sample included 34 men and 13 women whose mean age was 56.3 years (for details, see Figure 2), and whose mean BMI was 34.1 kg/m^2^.

Eight patients suffered from moderate OSA, whereas thirty-nine patients suffered from severe OSA, as defined by the AHI. The mean airway pressure of the CPAP they received was 10.3 cm of water pressure (6–18 cm H_2_O). For details, see Table 1.

### 2.2. Methods

The diagnosis of OSA was determined by using the ICSD-3 diagnostic criteria; the sleep testing was performed using a NOX A1 (Resmed Inc., San Diego, CA, U.S.) or a MiniScreen Plus (Saegeling Medizintechnik GmbH, Heidenau, Germany). The CPAP titration was performed using machines from the The AirSense 10 series (Resmed Inc., San Diego, CA, U.S.) or a Philips Dreamstation (Saegeling Medizintechnik GmbH, Heidenau, Germany). The indication for positive airway therapy was determined using the criteria defined by the guidelines of the Czech Sleep Research and Sleep Medicine Society [27]. CPAP therapy is recommended for patients with moderate to severe OSA (AHI/RDI ≥ 15).

Before having the patient use the machine, subjective nasal patency was measured using a visual analogue scale (VAS). The objective nasal patency was measured by a flowmeter (Elmet s.r.o., Přelouč, Czech Republic). Respiration data were analyzed, and the mean value of the inspirational peaks was evaluated. A pathological and clinically significant decrease in flow was defined as a flow of 4.57V or lower [28]. 

Nasal endoscopy was performed, and anatomical abnormalities that might cause nasal obstruction were recorded; our assessment was based on work published by Mladina et al. in 1987 and 2015 [29,30]. Each side of the nasal cavity was divided into one of six groups (endoscopic score ES6, see Table 2), and an ES6 score of 4 or more was defined as pathological and clinically significant for causing nasal obstruction [28]. 

After three months of CPAP treatment, the patients filled out a Sinonasal Outcome Test [31,32]. For the purposes of this study, questions targeting sleep and emotions were excluded. A score between 0 and 40 was calculated for each questionnaire. A score of 14.5 or more was taken to indicate a significant nasal intolerance [33]. 

The data of CPAP therapy adherence were evaluated after 12 months of CPAP treatment. 

### 2.3. Statistics

Statistical analyses were performed using NCSS 2021 Statistical Software (2021), NCSS, LLC. Kaysville, Utah, USA, ncss.com/software/ncss. The groups’ characteristics were described. Non-parametric tests were used for the subsequent analyses of the quantitative variables. A comparison of the quantitative values between groups according to compliance was performed. The hypothesis of agreement was tested against the alternative of disagreement. A two-sample t-test and non-parametric Mann–Whitney and Kolmogorov–Smirnov tests were used. The hypothesis of independence was tested in a contingency table against the alternative of dependence to compare genders between the groups. The Fisher’s exact test was used. *p* values < 0.05 were considered statistically significant.

## 3. Results

Of a total of forty-seven patients, only twelve patients showed low compliance to CPAP therapy after 12 months of treatment. The failure of CPAP therapy was statistically independent of all the measured variables: gender (*p* = 0.713), age (*p* = 0.427), BMI (*p* = 0.621), AHI (*p* = 0.124), ODI (*p* = 0.495), T90 (*p* = 0.502), basal saturation (*p* = 0.066), mean CPAP pressure (*p* = 0.057). For details, see Table 3. 

**Figure 3:**
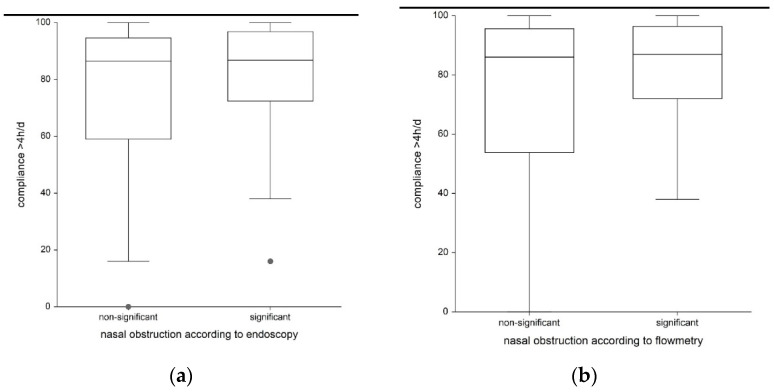
Relationship between CPAP compliance and nasal obstruction according to the endoscopy (**a**) and flowmetry (**b**) results..

**Figure 4:**
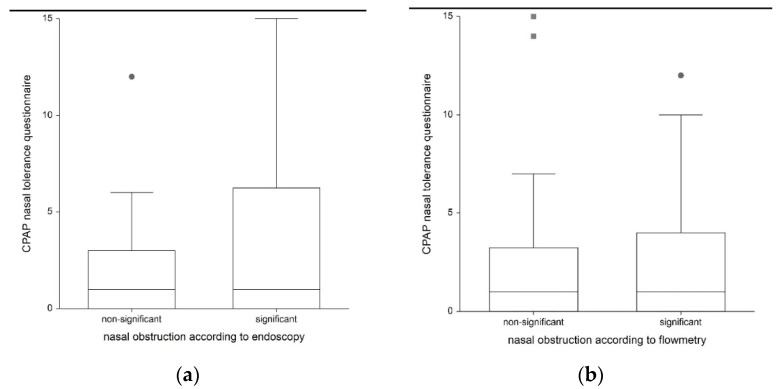
Relationship between the CPAP nasal tolerance questionnaire and nasal obstruction according to the endoscopy (**a**) and flowmetry (**b**) results..

The use of the ES6 yielded a score of 4 or more in at least one side of the nasal cavity in 20 patients. These 20 patients had a mean compliance of 79.0 % and had a mean score of 3.7 points on the questionnaire for CPAP nasal tolerance. The remaining 27 patients were without significant nasal obstruction, as shown by an endoscopy (an ES6 score of less than 4) and had a mean compliance of 73.6 % and a mean score of 1.9 points on the questionnaire for CPAP nasal tolerance. The difference between the groups was not statistically significant for either parameter (*p* = 0.498 for compliance, *p* = 0.588 for the questionnaire). For details, see Table 4 and Figure 3 and Figure 4.

Twenty-one patients had a significant decrease in flow measurement (less than 4.57 V measured with the flowmeter) in at least one side of their nasal cavity. These patients had a mean compliance of 79.7% and a mean of 2.9 (±3.8 SD) points scored on the CPAP nasal tolerance questionnaire. No significant flow drop was observed in twenty-six patients. These patients had a mean compliance of 72.9% and a mean of 2.5 (±4.0 SD) points scored on the CPAP nasal tolerance questionnaire. The difference between the groups was not statistically significant for either parameter (*p* = 0.754 for compliance, *p* = 0.657 for questionnaire). For details, see Table 4 and Figure 3 and Figure 4.

## 4. Discussion

Unobstructed upper airways are an important condition for an uncomplicated PAP treatment of OSA [15,18,24]. In some nasal obstruction cases, we can provide treatment by applying PAP through a full-face mask, but compliance is higher when the patients use a nasal mask [34,35,36]. 

Balsalobre et al. compared patients with nasal polyps to otherwise healthy subjects; both groups underwent CPAP. The control group showed a significant worsening of the nasal obstruction symptoms, as measured by VAS and the NOSE questionnaire (*p* < 0.01), and a significant decrease in nasal patency, as measured by the peak nasal inspiratory flow and acoustic rhinometry (*p*  <  0.01) [37].

Our research was aimed at revealing the relationship between CPAP adherence and nasal obstruction, as assessed and evaluated by nasal endoscopy (ES6) and nose cavity flow measurement [28]. We employed a strict exclusion process: only 47 out of 450 patients met the demands of the inclusion criteria. For details, see the flowchart in Figure 1. 

The final sample of our study represented the real-world population: 13 female and 34 male patients with mean a BMI of 35.4 and 33.6 and a mean AHI of 40.0 and 55.2, respectively. CPAP failure was associated neither with demographic data such as gender (*p* = 0.713), age (0.427) or BMI (*p* = 0.621), nor with sleep-monitoring data, such as AHI (*p* = 0.124) or T90 (*p* = 0.502).

The guidelines of the surgical division of the Czech Sleep Research and Sleep Medicine Society [38] were complied with based on the premise that nasal surgery does not significantly affect obstructive respiratory events [39,40]. This assumption is based on the fact that only 16.7% of the patients with OSA who undergo nasal surgery meet the Sher criteria [19]. Several works present findings that indicate that a reduction in obstructive respiratory events can be achieved by intranasal corticosteroids application, particularly in children and allergic individuals [41,42]. According to two pooled randomized, placebo-controlled clinical trials, nasal steroids where shown to improve CPAP adherence—the studies recorded an overall 0.4 h per night increase in the usage of the machine. However, this increase did not reach statistical significance (*p* = 0.19). There was no increase in the percentage of nights during which CPAP was used, nor was there a significant difference in nasal symptoms [43]. 

Nasal surgery can positively influence subjective sleep parameters, e.g., snoring or excessive daytime sleepiness [19,44,45], and nasal patency improvement can be useful for reducing the level of PAP when treating OSA, resulting in better adherence to CPAP therapy [46,47]. However, the positive effect of surgery on CPAP tolerance was not confirmed in all the published literature [48]. Therefore, prudence should be applied when selecting candidates for nasal interventions. 

Nasal procedures with the aim of improving nasal patency, e.g., septoplasty, are frequently performed. These operations carry only a low risk with low morbidity and a low rate of complications; we rarely encounter complications such as the formation of nasal septal perforation or nasal synechiae [49,50]. Van Egmond et al. compared the effectiveness of septoplasty combined with turbinate surgery with the efficacy of septoplasty on its own in the treatment of nasal obstruction due to a deviated nasal septum. Subjective and objective outcomes generally appeared to improve after the treatment. However, the additional benefit of turbinate surgery was not evident. Moreover, the subjective benefit was not always accompanied by an objective improvement, and vice versa. Despite the routine application of septoplasty in clinical practice, the body of evidence does not support firm conclusions on its effectiveness [49]. Nasal packing after septoplasty was even more likely to cause adverse events, including respiratory distress, pain, sleep disturbance, crusting, epiphora, dysphagia and adhesion. Routine nasal packing after septoplasty should therefore be avoided [51]. 

In general, isolated nasal treatments are the least effective in treating OSA, but multi-level surgery may provide an alternative to CPAP treatment [52]. The effect of multi-level surgery (multiple surgeries to include nasal surgery, tonsillectomy, palate surgery, pharyngeal surgery and tongue surgery) on OSA was evaluated by Lin et al., who observed a reduction in the apnea hypopnea index in 1978 patients. They recorded a reduction of 29 events per hour: from 48.0 to 19.0 events/hour (a 60.3% reduction, *p*-value < 0.0001) [53]. 

Nasal surgery may be offered to CPAP-intolerant patients—adjunctive nasal surgery may facilitate improved postoperative CPAP adherence due to lower CPAP requirements (average of 2–3 cm H_2_O) or improved tolerance of nasal-type masks without the necessity of a chin strap [54]. 

Kempfle et al. concluded that nasal surgery (septoplasty or turbinate reduction) would be a cost-effective way to increase CPAP adherence. In the short term, septoplasty surgery was not a cost-effective way to improve CPAP adherence in patients who had a great baseline difficulty using CPAP, but over a longer time span of 10 or 15 years, septoplasty became increasingly more cost-effective. The cheaper turbinate reduction would be a cost-effective way to increase CPAP adherence regardless of the time span in question. Notably, perioperative and postoperative surgical complications did not unfavorably influence the cost-effectiveness of either surgery. Therefore, surgical intervention for non-adherent CPAP users, or partially adherent CPAP users, should be considered a part of a multifaceted approach to improve CPAP adherence [55].

CPAP adherence and endoscopic findings assessed by ES6 (modified Sinonasal Outcome Test), as well as the objective assessment of air flow by a flowmeter were shown to be independent of each other in our group. 

We compared our results to those of similar studies published in 2019 by Inoue et al. [21] and in 2017 by Park et al. [56]. While Inoue et al. studied a larger sample (*n* = 543), they reported the same results in their long-term follow up (more than 1 year). Their short-term results (less than 1 year follow up), however, differed. Park et al. used a cohort whose size was similar to that of ours and, like us, they concluded that CPAP non-adherence was independent of sleep parameters (*p* = 0.671). Their results differed in the evaluation of nose patency influence (*p* ≤ 0.0001). Our conclusions concerning the nasal flow measurement are in agreement with a systematic review authored by Brimioulle and Chaidas in 2022 [57] but in conflict with the study by Sugiura et al. from 2007, which was performed on a similar sample (*n* = 51). Sugiura’s work [24] concluded that nasal obstruction is a significant factor for CPAP non-adherence (*p* = 0.002) but, surprisingly, showed the same association with the AHI (*p* = 0.003). We attach more reliability to the systematic review by Brimioulle because they evaluated 63 works and pointed out some contradictory results. 

The limits of our study are its monocentricity and a strict exclusion process (47 out of 450 patients were enrolled). However, the studied sample represents the real-world population with OSA. For details, see Figure 1 and Table 1. 

## 5. Conclusions

Our study demonstrated independence between CPAP adherence and endoscopic findings/nasal patency. Our data show that although the endoscopic findings in the nasal cavity could indicate an obstruction, compliance to CPAP therapy was not reduced, and neither was it reduced with a decrease in nasal flow, according to our observation. 

## Figures and Tables

**Table 1:** Input data of studied sample.

Group	Female					Male					Total				
	*n* = 13					*n* = 34					*n* = 47				
	Mean	SD	Median	IQR	Range	Mean	SD	Median	IQR	Range	Mean	SD	Median	IQR	Range
Age (years)	61.6	17.7	61.5	18	49–74	54.2	26.9	55.0	16.8	36–74	56.3	10.7	56	17.0	36–74
BMI (kg/m2)	35.4	5.1	36.5	7.4	27.8–44.4	33.6	7.5	33.5	6.2	25.7–44.7	34.1	7.0	34.3	6.8	25.7–44.7
AHI (n/h)	40.0	34.2	43.4	22.9	18.6–119.2	55.2	26.8	57.9	33.0	18.5–95.8	51.0	23.7	47.4	36.1	18.5–119.2
30 > AHI ≥ 15(n)	4		30.8%			4		11.8%			8		17.0%		
AHI ≥ 30 (n)	9		69.2%			30		88.2%			39		83.0%		
ODI (n/h)	40.3	26.4	31.7	22.6	18.5–118.7	51.9	23.0	55.1	36.3	16.0–116.0	48.6	24.2	45.2	34.0	16.0–118.7
Basal O2 sat. (%)	90.9	3.4	92.0	2.5	83.0–95.0	90.5	16.0	92.0	5.0	79.0–96.0	90.6	13.7	92.0	5.0	79.0–96.0
Average low O2sat. (%)	85.6	4.2	87.0	5.5	77.0–92.0	84.1	15.9	86.0	8.5	65.0–92.0	84.5	13.7	86.5	8.0	65.0–92.0
T 90 (%)	19.8	18.9	10.3	22.4	0.7–63.6	29.7	25.0	26.2	46.6	1.0–69.3	26.9	23.6	14.9	43.0	0.7–69.3
Mean CPAP (cm H2O)	10.5	1.8	10.6	2.0	6.0–13.0	10.1	3.1	10.0	3.75	6.0–18.0	10.3	2.8	10.0	3.0	6.0–18.0

**Table 2:** Endoscopic score ES6.

Group	Septal Deformity	Inferior Turbinate Hypertrophy
1	No	No
2	No	Yes
3	Non-significant (type 1,3,6)	No
4	Non-significant (type 1,3,6)	Yes
5	Significant (type 2,4,5)	No
6	Significant (type 2,4,5)	Yes

**Table 3:** Comparison of the CPAP compliant and CPAP non-compliant patient groups.

Group	Compliant					Non-Compliant					p
	*n* = 35					*n* = 12					
	Male			Female		Male			Female		
Gender	28	74.3%		7	25.7%	8	66.7%		4	33.3%	0.713
	Mean	SD	Median	IQR	Range	Mean	SD	Median	IQR	Range	
Age (years)	55.7	11.1	54.0	18	36–74	57.8	9.7	56.5	11.8	36–72	0.427
BMI (kg/m2)	34.3	7.7	34.7	7.6	25.7–44.7	33.5	4.2	32.8	6.3	28.3–43.2	0.621
AHI (n/h)	54.1	24.3	52.0	36.2	18.5–119.2	41.9	19.7	33.6	37.8	18.6–71.8	0.124
30 > AHI ≥ 15(n)	4		11.4%			4		33.3%			
AHI ≥ 30 (n)	31		88.6%			8		66.7%			
ODI (n/h)	50.4	26.4	45.6	37.9	16.0–118.7	43.6	16.4	41.4	23.2	18.6–64.4	0.495
Basal O2 saturation (%)	90.1	15.7	91.0	5.3	79.0–96.0	92.1	2.7	93.0	2.8	86.0–95.0	0.066
Average low O2 sat. (%)	84.2	15.5	86.0	8.0	70.0–92.0	85.5	5.7	87.5	88.0	75.0–92.0	0.501
T 90 (%)	28.4	24.0	23.2	45.3	1.0–69.3	22.6	23.0	11.2	38.8	0.7–65.8	0.502
Mean CPAP (cm H20)	9.9	2.1	10.0	2.4	6.0–13.5	11.4	3.0	10.7	3.8	8.0–18.0	0.057

**Table 4:** Compliance to CPAP therapy in relation to ES6 and flow measurement.

ES6		≥4					<4					*p*
		N = 20					N = 27					
		Mean	SD	Median	IQR	Range	Mean	SD	Median	IQR	Range	
	CPAP compl.	79.0%	23.5	85.7	24.3	16.0–100.0	73.6 %	26.8	86.4	35.6	0.0–100.0	0.498
	questionnaire	3.7	5.0	1.0	6.3	0.0–15.0	1.9	2.7	1.0	3.0	0.0–12.0	0.588
Flow measurement		<4.57 V					≥4.57 V					
		N = 21					N = 26					
	CPAP compl.	79.7 %	18.1	87.0	24.4	38.0–100.0	72.9 %	29.9	86.1	41.7	0.0–100.0	0.754
	questionnaire	2.9	3.9	1.0	4.0	0.0–12.0	2.5	4.0	1.0	3.3	0.0–15.0	0.657

## Data Availability

The data presented in this study are available on request from the corresponding author. The data are not publicly available due to privacy and ethical restrictions.

## Abbreviations

AHI	apnea-hypopnea index
BMI	body mass index
CPAP	continuous positive airway pressure
ES6	endoscopic score 6
ICSD-3	international classification of sleep disorders—third edition
OCST	out-of-center sleep testing
ODI	oxygen desaturation index
OSA	obstructive sleep apnea
PAP	positive airway pressure
PG	polygraphy
PSG	polysomnography
VAS	visual analogue scale
